# The association between a body shape index and depressive symptoms: a cross-sectional study using NHANES data (2011–2018)

**DOI:** 10.3389/fnut.2024.1510218

**Published:** 2025-01-24

**Authors:** Zheng Zhang, Xiang-Yan Ruan, Wei Ma

**Affiliations:** ^1^Department of Sports Science, Kyonggi University, Suwon, Republic of Korea; ^2^College of Martial Arts, Hebei Sports College, Shijiazhuang, China

**Keywords:** a body shape index, depressive symptoms, crosssectional analysis, NHANES, association

## Abstract

**Objective:**

This study aimed to explore the relationship between A Body Shape Index (ABSI) and depressive symptoms, and to evaluate the moderating effects of sociodemographic, lifestyle, and health factors.

**Methods:**

We analyzed data from 19,659 participants in the 2011–2018 National Health and Nutrition Examination Survey (NHANES). Depressive symptoms were assessed using the Patient Health Questionnaire-9 (PHQ-9). ABSI was calculated from waist circumference, height, and BMI. Multiple linear regression models were employed to investigate the association between ABSI and depressive symptoms, with subgroup analyses to examine potential associations within specific populations.

**Results:**

Higher ABSI was significantly associated with increased depressive symptoms, after adjusting for covariates including age, sex, race, marital status, poverty-income ratio, smoking, alcohol consumption, physical activity, diabetes, and hypertension. Individuals in the highest ABSI quartile (Q4) had significantly more depressive symptoms compared to those in the lowest quartile (Q1) (*β* = 0.22, 95% CI = 0.02–0.41, *p* = 0.0323). Subgroup analyses revealed that marital status and hypertension significantly moderated the association between ABSI and depressive symptoms.

**Conclusion:**

This study provides the first comprehensive analysis of the link between ABSI and depressive symptoms, suggesting that higher ABSI is associated with greater depressive symptoms. These findings highlight the potential importance of waist circumference and abdominal fat distribution in assessing depression risk. Future research should explore the causal mechanisms underlying this association and investigate the biological pathways involved, to inform more effective strategies for depression prevention and intervention.

## 1 Background

Depression is one of the most prevalent mental health disorders globally, profoundly affecting individuals’ quality of life, social functioning, and physical health (1). The World Health Organization (WHO) estimates that over 264 million people worldwide suffer from depression. Characterized by persistent low mood, loss of interest, fatigue, and diminished self-esteem, depression not only increases the risk of cardiovascular and metabolic diseases but is also closely linked to higher mortality rates (2–4). As a leading contributor to the global burden of disease, depression remains a critical public health concern. While its etiology is complex, involving genetic, environmental, and psychosocial factors, growing evidence suggests that body shape, particularly fat distribution, may play an important role in the predisposition to and development of depression (5, 6).

The A Body Shape Index (ABSI) has garnered increasing attention in recent years as a novel measure of body shape and its associated health risks (6). Traditionally, Body Mass Index (BMI) has been the standard for assessing obesity and related risks. However, BMI cannot differentiate between muscle mass and fat, and it fails to account for fat distribution. Similarly, waist circumference, while widely used as a measure of central obesity, has its limitations. It does not account for differences in body size or proportions, and higher waist circumference in taller individuals may not necessarily indicate increased health risks. These limitations reduce the specificity of waist circumference as a standalone measure of body composition and health outcomes. In contrast, ABSI incorporates waist circumference, height, and BMI to emphasize the independent contribution of abdominal fat to health outcomes (7). Abdominal fat, particularly visceral fat, is strongly linked to metabolic abnormalities, chronic inflammation, and endocrine dysregulation—factors that may also serve as biological triggers for depression (8). This growing understanding underscores the need to further investigate the relationship between body fat distribution and mental health.

The relationship between body shape and mental health, particularly the link between body fat distribution and depressive symptoms, has attracted growing research interest. An expanding waist circumference is not only associated with a heightened risk of metabolic disorders but may also adversely affect mental health by impacting self-image, social interactions, and biological pathways such as inflammation and hormonal dysregulation (9). As a specific indicator of abdominal adiposity, ABSI may play a key role in assessing the risk of depression. However, research on the association between ABSI and depressive symptoms remains limited, and the precise mechanisms linking the two are yet to be fully understood. To address this gap, the present study aimed to explore the association between ABSI and depressive symptoms, while examining how sociodemographic, lifestyle, and health-related factors contribute to this relationship. By analyzing data from a large population sample, we aim to provide fresh perspectives and evidence to deepen the understanding of the complex interplay between body image and mental health.

While numerous studies have explored the relationship between body type and mental health, particularly the link between obesity and depression, most continue to rely on Body Mass Index (BMI) as the primary indicator of body composition (10–13). Although BMI is widely used in epidemiological research, it has several limitations. First, BMI cannot distinguish between muscle mass and fat mass, leading to potential misclassification of obesity in individuals with high muscle mass but low body fat (14). Second, BMI does not capture the specific distribution of body fat, particularly waist circumference, a key indicator of abdominal adiposity that is closely linked to metabolic disease, chronic inflammation, and endocrine dysfunction (15, 16). These factors, critical to health outcomes, are not fully accounted for by BMI alone. To address these limitations, the A Body Shape Index (ABSI) has been proposed as a novel metric for body shape assessment. ABSI offers a more refined measurement by incorporating waist circumference, height, and BMI, thereby better reflecting the independent contribution of abdominal fat to health risks. It has been shown to predict metabolic abnormalities, cardiovascular disease, and all-cause mortality more accurately than BMI. Despite its potential value in predicting physical health outcomes, however, research on the relationship between ABSI and mental health remains limited. This gap leaves an incomplete understanding of the connection between body shape and mental health, particularly in assessing depression risk. Whether ABSI provides unique predictive insights into mental health outcomes remains unclear.

Studies investigating the relationship between body shape and depression have often yielded conflicting results. Some have reported a positive association between obesity and depression (17), while others have found no significant link (18), with some even suggesting that obesity may be associated with a lower risk of depression at certain life stages (19). These inconsistencies may stem from the different body shape indicators used. For example, studies relying on BMI may overlook the impact of abdominal fat on depressive symptoms. Specific indicators of abdominal obesity, such as waist circumference, may provide more relevant insights into these associations (20). As such, exploring the relationship between ABSI and depression could not only address the limitations of BMI but also offer a more refined tool for assessing body shape and its mental health implications. This could open new perspectives on the multidimensional risk factors for depression. In this context, the aim of the present study is to investigate the potential association between body shape, as measured by ABSI, and depressive symptoms, while examining the influence of sociodemographic, lifestyle, and health factors on this relationship. By addressing this research gap, we hope to provide new evidence on the impact of body shape—particularly abdominal fat distribution—on depression. Our findings could form the basis for future clinical assessments and interventions aimed at mitigating the mental health risks associated with body fat distribution.

## 2 Methods

### 2.1 Study population

**Figure 1:**
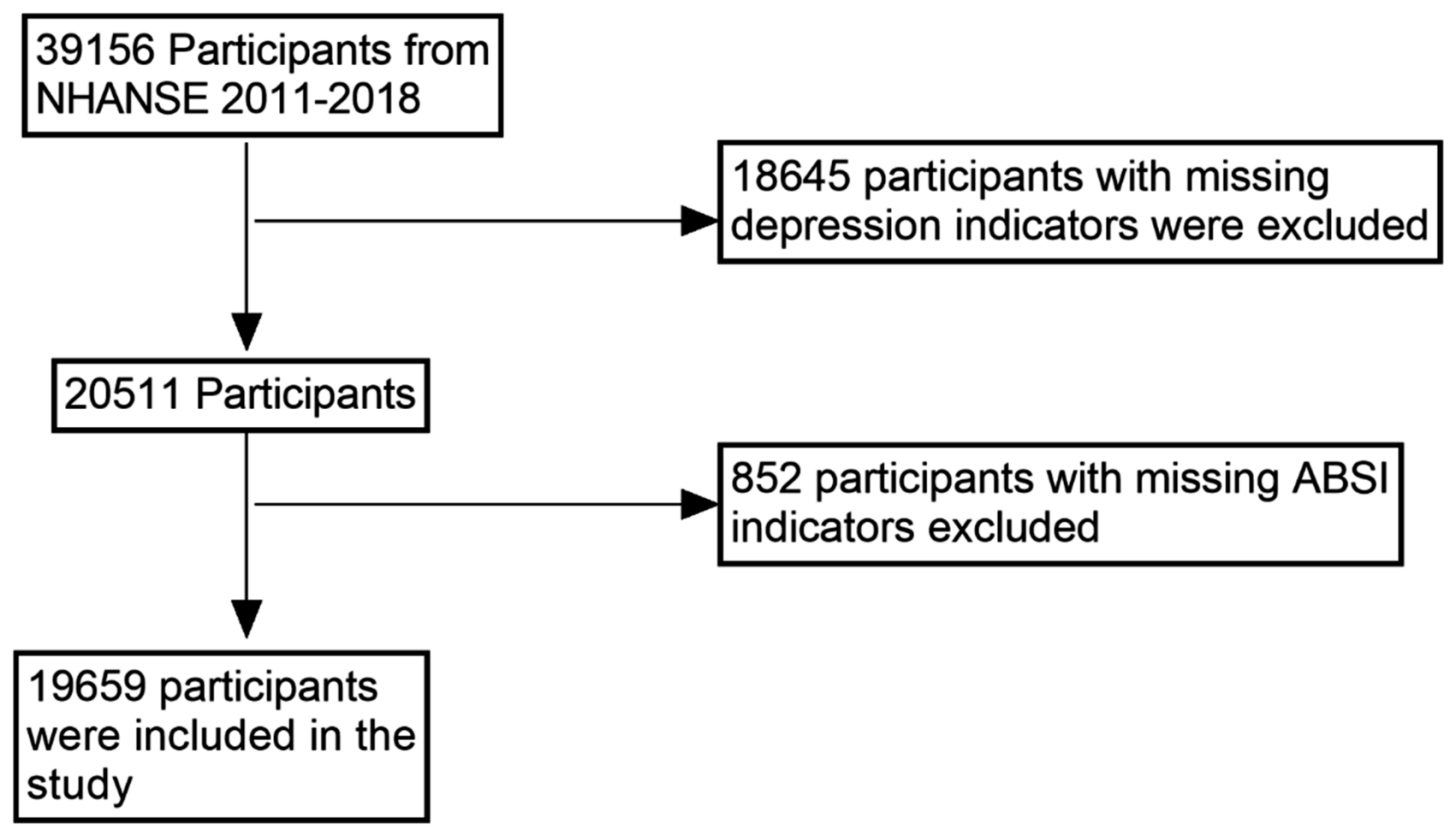
Flowchart.

This study utilized data from the 2011–2018 National Health and Nutrition Examination Survey (NHANES), a nationally representative, multistage, stratified survey designed to assess the health and nutritional status of adults and children in the United States. Conducted by the Centers for Disease Control and Prevention (CDC), NHANES provides data that are widely extrapolated to represent the health status of the non-institutionalized US population. The original dataset included 39,156 participants. During data cleaning, 18,645 individuals were excluded due to missing depression scores (PHQ-9 data), and an additional 852 participants were excluded due to incomplete body shape index (ABSI) data. Ultimately, a total of 19,659 participants were included in the final analysis (see Figure 1). This sample had complete information on key covariates, including marital status, physical activity, alcohol consumption, smoking, diabetes, poverty income ratio (PIR), and hypertension.

### 2.2 Measurement of depression levels

Depression levels in this study were assessed using the Patient Health Questionnaire-9 (PHQ-9), a widely used self-report tool in clinical and epidemiological research for measuring the severity of depressive symptoms. Participants rated nine items—including low mood, sleep disturbances, fatigue, and self-reported mood and behavioral functioning over the past 2 weeks—on a scale of 0 to 3, where 0 corresponds to “never” and 3 to “almost every day.” The total PHQ-9 score ranges from 0 to 27. Depressive symptoms were categorized based on the total PHQ-9 score: 0–4 as no depressive symptoms, 5–9 as mild depression, 10–14 as moderate depression, 15–19 as moderately severe depression, and 20 and above as severe depression (21). For the purposes of this study, the PHQ-9 score was treated as a continuous variable to capture the full spectrum of depressive symptom severity and assess its potential association with ABSI. The PHQ-9 was chosen as the primary outcome measure in this study due to its ability to comprehensively reflect the breadth and severity of depressive symptoms. Its simplicity and ease of use make it an ideal tool for epidemiological research (22).

### 2.3 Measurement of ABSI

A Body Shape Index (ABSI) is a novel metric for assessing body shape and health risk, offering a more refined prediction of health outcomes by integrating waist circumference, height, and weight (23). Unlike the traditional Body Mass Index (BMI), which does not account for fat distribution, ABSI evaluates health risks by considering the relative proportions of waist circumference to other body dimensions. By employing isochronous growth regression, ABSI addresses the limitations of BMI, particularly its failure to capture the risks associated with abdominal fat. Research has demonstrated that higher ABSI values are strongly correlated with increased mortality from various chronic conditions, such as cardiovascular disease and diabetes, as well as all-cause mortality. As a result, ABSI serves as a more precise tool for assessing health risks related to body shape. In this study, ABSI was treated as a continuous variable to evaluate its potential relationship with depressive symptoms, as measured by PHQ-9 scores. The formula for ABSI is as follows:

$$\mathrm{ABSI}=\left(\mathrm{Waist Circumference}\left(m\right)\right)/\left(BMI2/3\times \mathrm{Height}\left(m\right)1/2\right)$$

### 2.4 Assessment of covariates

To control for potential confounders and ensure an accurate assessment of the association between A Body Shape Index (ABSI) and depressive symptoms (measured by the PHQ-9 score), several covariates were included in this study. These covariates were selected based on theoretical relevance and empirical evidence from prior studies, encompassing sociodemographic, lifestyle, and health factors. Each covariate was adjusted to mitigate potential confounding effects on the observed associations.

#### 2.4.1 Sociodemographic factors

- Age and sex:
  Age and sex were included as fundamental demographic variables due to their well-established associations with both body composition and depressive symptoms (24). For example, age has been linked to changes in body fat distribution and depressive symptom prevalence, while sex differences influence both body shape and mental health outcomes. Age was treated as a continuous variable, while sex was included as a dichotomous variable (male, female).
- Race/ethnicity:
  Race/ethnicity was included to account for potential differences in genetic, cultural, and socioeconomic factors that may influence ABSI and depressive symptoms. Self-reported race/ethnicity was categorized into five groups: Non-Hispanic White people, Non-Hispanic Black people, Mexican American, other Hispanic people and other race/ethnicity.
- Marital status:
  Marital status was selected due to its potential impact on social support and mental health. Studies have shown that individuals who are married or cohabiting may experience better mental health outcomes compared to those who are widowed, divorced, separated, or never married (25). Marital status was categorized into three groups: married or cohabiting, widowed/divorced/separated, and never married.
- Poverty income ratio (PIR):
  PIR serves as a proxy for socioeconomic status, which is a known determinant of both physical and mental health (26). Low income has been associated with higher stress levels and limited access to healthcare, potentially influencing depressive symptoms. PIR was categorized into three groups: low income (PIR < 1), medium income (1 ≤ PIR < 2), and high income (PIR ≥ 2).
- Educational attainment:
  Educational attainment reflects an individual’s socioeconomic and health literacy status, which can influence health behaviors and mental health. This variable was categorized into five levels: less than primary school, middle school, high school graduate/GED, some college or associate’s degree, and college graduate or higher.

#### 2.4.2 Lifestyle factors

- Smoking status:
  Smoking behavior was included as a potential confounder because smoking has been linked to both body composition and mental health outcomes, including depressive symptoms (27). Smoking status was categorized as never smoker, ever smoker, and current smoker based on self-reports.
- Drinking status:
  Alcohol consumption can influence both physical health and mental well-being (28). Participants’ drinking history was categorized into never, ever, and current drinkers to account for its potential confounding effects.
- Physical activity:
  Physical activity was considered due to its well-documented effects on body composition and mental health (29). Self-reported leisure-time physical activity was used to classify participants into four categories: inactive, moderately active, vigorously active, or both moderately and vigorously active. Classification was based on responses to two NHANES questionnaire items: Moderate-intensity activities: Participants were asked, “In a typical week, do you do any moderate-intensity sports, fitness, or recreational activities that cause a small increase in breathing or heart rate such as brisk walking, bicycling, swimming, or volleyball for at least 10 min continuously? “Vigorous-intensity activities: Participants were asked, “In a typical week, do you do any vigorous-intensity sports, fitness, or recreational activities that cause large increases in breathing or heart rate like running or basketball for at least 10 min continuously? “Participants who answered “no” to both questions were classified as inactive. Those who answered “yes” only to moderate-intensity activities were classified as moderately active, and those who answered “yes” only to vigorous-intensity activities were classified as vigorously active. Participants who answered “yes” to both questions were classified as both moderately and vigorously active.

#### 2.4.3 Health factors

- Diabetes and hypertension status:
  Chronic health conditions like diabetes and hypertension were included because they can influence both body composition and depressive symptoms, potentially confounding the association between ABSI and depressive symptoms. These variables were treated as dichotomous (present or absent) based on self-reports or physician diagnoses.

#### 2.4.4 Consideration of residual confounding

Although this study included a comprehensive set of covariates, residual confounding factors might still exist. For instance:

- Unmeasured psychological variables: Variables such as personality traits, stress levels, and access to mental health resources were not included but may influence depressive symptoms.
- Dietary habits: Detailed dietary patterns were not adjusted due to data limitations, which might affect both body shape and mental health.
- Genetic predisposition: Genetic factors influencing body shape and mental health outcomes were not accounted for in this analysis.

These unmeasured factors may have introduced residual confounding. Future studies incorporating more granular data on psychological, dietary, and genetic factors are needed to validate and refine the findings of this study.

### 2.5 Statistical analysis

All analyses were conducted using Stata software (Stata Corp, College Station, TX). The analyses accounted for the complex sampling design of the NHANES data, including stratification, clustering, and weighting, to ensure that the results were nationally representative. All *p*-values were two-tailed, with statistical significance set at *p* < 0.05. First, descriptive statistics were performed for all variables. Means and standard deviations were reported for continuous variables, while frequencies and percentages were presented for categorical variables. Analysis of variance (ANOVA) and chi-squared tests were used to evaluate initial associations between the four ABSI subgroups and PHQ-9 scores. Multiple linear regression models were employed to assess the association between ABSI and depressive symptoms (PHQ-9 scores) in three steps:

1. Unadjusted model: This model examined the direct association between ABSI and PHQ-9 scores without adjusting for covariates.
2. Adjusted Model I: This model controlled for sociodemographic variables, including age, sex, and race/ethnicity.
3. Adjusted Model II: This fully adjusted model controlled for all covariates, including marital status, educational attainment, poverty income ratio (PIR), smoking status, alcohol consumption, physical activity, diabetes, and hypertension.

Subgroup analyses further explored the association between ABSI and depressive symptoms within specific populations. These analyses, fully adjusted for all covariates, stratified the association by sex and age to identify potential group-specific trends or idiosyncratic effects. Additionally, smoothed curve fitting was employed to explore potential non-linear associations between ABSI and PHQ-9 scores, allowing for the identification of more complex patterns. All analyses were adjusted for NHANES sample weights to ensure that the results could be generalized to the U.S. adult population.

## 3 Results

The findings are presented across three key tables, each addressing distinct aspects of the analysis:

- Table 1 summarizes the baseline characteristics of the study population, including depressive symptoms (PHQ-9 scores) and ABSI, stratified by ABSI quartiles. These descriptive statistics provide an overview of the sample distribution and highlight group-level differences.
- Table 2 details the association between ABSI quartiles and depressive symptoms, assessed using linear regression models. This analysis examines the overall relationship, focusing on the population-wide impact of ABSI.
- Table 3 presents subgroup analyses, exploring the association between ABSI (as a continuous variable) and depressive symptoms within specific subgroups. These analyses aim to identify potential interaction effects, offering a more granular understanding of variations across demographic and clinical characteristics.

**Table 1:** Characteristics of study population.

MET	Q1	Q2	Q3	Q4	*P*-value
N	4,922	4,922	4,922	4,923	
Age (years)	35.53 ± 14.40	43.08 ± 15.49	49.31 ± 15.75	59.17 ± 15.28	<0.0001
Standing height (cm)	167.79 ± 9.84	168.73 ± 9.95	169.04 ± 10.03	168.64 ± 10.29	<0.0001
BMI, (kg/m^2^)	28.51 ± 7.60	28.96 ± 6.90	29.64 ± 6.86	29.53 ± 6.21	<0.0001
Waist Circumference (cm)	90.29 ± 15.25	97.28 ± 15.39	102.92 ± 15.97	108.31 ± 15.48	<0.0001
PIR	2.88 ± 1.69	3.07 ± 1.66	3.07 ± 1.65	2.89 ± 1.62	<0.0001
Gender (%)					<0.0001
Male	43.35%	48.57%	52.86%	51.83%	
Female	56.65%	51.43%	47.14%	48.17%	
Race					<0.0001
Mexican American	8.71%	9.99%	9.80%	6.27%	
Other Hispanic people	7.56%	6.48%	6.44%	4.37%	
Non-Hispanic White People	56.18%	63.61%	66.90%	75.34%	
Non-Hispanic Black people	18.48%	10.77%	8.53%	6.45%	
Other Race - Including Multi-Racial	9.06%	9.15%	8.32%	7.56%	
Education level					<0.0001
Less than 9th grade	2.62%	3.56%	4.92%	5.99%	
9–11th grade	7.53%	8.48%	9.88%	10.56%	
High school graduate/GED or equivalent	20.94%	20.92%	23.56%	25.05%	
Some college or AA degree	34.16%	32.82%	31.75%	31.01%	
College graduate or above	34.75%	34.19%	29.89%	27.35%	
Marital status					<0.0001
Married	44.60%	55.10%	58.94%	58.09%	
Widowed	1.75%	3.32%	4.97%	11.02%	
Divorced	7.69%	9.74%	11.16%	12.80%	
Separated	2.36%	2.76%	2.10%	2.39%	
Never married	32.47%	19.32%	14.86%	9.80%	
Living with partner	11.11%	9.74%	7.96%	5.87%	
Smoking status					<0.0001
Never smoking	66.70%	60.81%	52.53%	45.29%	
Former smoking	15.57%	19.80%	27.23%	34.54%	
Current smoking	17.72%	19.39%	20.24%	20.17%	
Alcohol status					<0.0001
Never drinking	12.68%	10.01%	10.63%	10.55%	
Former drinker	2.52%	3.23%	3.24%	5.08%	
Current drinker	84.80%	86.76%	86.13%	84.37%	
Physical activity					<0.0001
Inactive	32.43%	41.14%	46.80%	56.73%	
Moderate	22.54%	26.18%	30.71%	31.26%	
Vigorous	14.74%	10.28%	6.19%	3.05%	
Both moderate and vigorous	30.29%	22.41%	16.31%	8.96%	
High blood pressure					<0.0001
Yes	17.97%	26.49%	35.02%	47.89%	
No	81.93%	73.45%	64.96%	51.93%	
Diabetes					<0.0001
Yes	3.39%	5.70%	11.95%	19.15%	
No	95.34%	92.19%	85.51%	77.19%	
Depression					0.0003
Yes	7.55%	7.42%	7.50%	9.45%	
No	92.45%	92.58%	92.50%	90.55%	

BMI, body mass index; PHQ-9, Patient Health Questionnaire-9.

**Table 2:** The association between a body shape index (ABSI) and depressive symptoms.

Model	Q1	Q2β (95% CI)	Q3β (95% CI)	Q4β (95% CI)
Non-adjusted		−0.06 (−0.22, 0.10) *p* = 0.4619	0.09 (−0.07, 0.26) *p* = 0.2700	−0.01 (−0.18, 0.16) *p* = 0.9034
Adjust I	Reference	0.13 (−0.03, 0.29) *p* = 0.1213	0.42 (0.25, 0.59) p < 0.0001	0.10 (−0.08, 0.28) *p* = 0.2577
Adjust II	Reference	−0.01 (−0.18, 0.16) p = 0.9034	0.87 (0.68, 1.06) p < 0.0001	0.22 (0.02, 0.41) *p* = 0.0323

Adjust I, Adjusted for Gender, Age and Race; Adjust II, Adjusted for Gender, Age, Race, Education level, Marital status, High blood pressure, Diabetes, Physical activity, Smoking status, Alcohol status, PIR.

**Table 3:** Subgroup analysis of the association between ABSI and depressive symptoms.

Character	Crude	Model IIβ (95%CI)	*P*	P _interaction_
Gender				0.6509
Male	Reference	29.24 (4.82, 53.65)	0.0189	
Female	Reference	22.16 (3.03, 41.30)	0.0232	
Race				0.8767
Mexican American	Reference	17.31 (−27.00, 61.63)	0.4438	
Other Hispanic people	Reference	3.85 (−45.45, 53.15)	0.8782	
Non-Hispanic White People	Reference	30.07 (6.18, 53.96)	0.0136	
Non-Hispanic Black people	Reference	31.14 (0.32, 61.95)	0.0477	
Other Race - Including Multi-Racial	Reference	31.15 (−9.42, 71.72)	0.1324	
Education level				0.4355
Less than 9th grade	Reference	12.02 (−45.16, 69.21)	0.6803	
9–11th grade	Reference	22.95 (−20.07, 65.98)	0.2958	
High school graduate/GED or equivalent	Reference	8.01 (−23.11, 39.13)	0.6139	
Some college or AA degree	Reference	46.01 (19.50, 72.52)	0.0007	
College graduate or above	Reference	23.04 (−7.42, 53.50)	0.1383	
Age				0.7608
<40	Reference	27.07 (1.34, 52.80)	0.0392	
≥ 40, <60	Reference	24.66 (−1.41, 50.73)	0.0637	
≥ 60	Reference	14.50 (−10.32, 39.32)	0.2523	
PIR				0.1776
<1	Reference	−1.38 (−33.20, 30.43)	0.9321	
≥ 1, <2	Reference	36.61 (7.72, 65.50)	0.0130	
≥ 2	Reference	29.67 (8.24, 51.11)	0.0067	
Alcohol status				0.6898
Never drinking	Reference	10.33 (−28.41, 49.07)	0.6012	
Former drinker	Reference	31.98 (−35.40, 99.36)	0.3522	
Current drinker	Reference	28.45 (11.48, 45.42)	0.0010	
Smoking status				0.1658
Never smoking	Reference	23.53 (3.73, 43.32)	0.0199	
Former smoking	Reference	9.04 (−23.54, 41.62)	0.5866	
Current smoking	Reference	53.40 (19.27, 87.54)	0.0022	
Physical activity				0.3750
Inactive	Reference	13.40 (−7.64, 34.44)	0.2119	
Moderate	Reference	45.37 (15.60, 75.14)	0.0028	
Vigorous	Reference	34.23 (−24.71, 93.18)	0.2550	
Both moderate and vigorous	Reference	28.64 (−9.67, 66.95)	0.1428	
Diabetes				0.7883
Yes	Reference	28.04 (−12.77, 68.85)	0.1781	
No	Reference	27.71 (11.13, 44.30)	0.0011	
High blood pressure				0.0417
Yes	Reference	5.89 (−18.63, 30.40)	0.6379	
No	Reference	38.20 (18.94, 57.45)	0.0001	
Marital status				0.0004
Married	Reference	13.31 (−8.61, 35.23)	0.2339	
Widowed	Reference	−17.26 (−68.90, 34.38)	0.5123	
Divorced	Reference	79.80 (34.78, 124.82)	0.0005	
Separated	Reference	145.26 (67.77, 222.74)	0.0002	
Never married	Reference	40.92 (6.78, 75.07)	0.0188	
Living with partner	Reference	−20.26 (−72.91, 32.39)	0.4508	

Together, these tables provide a comprehensive framework for understanding the relationship between ABSI and depressive symptoms, progressing logically from descriptive population characteristics to inferential analyses and subgroup-specific insights.

### 3.1 Baseline situation

A total of 19,659 participants were included in this study. Baseline characteristics revealed significant differences between groups when stratified by ABSI quartiles. As ABSI increased, the mean age rose from 35.53 years in the lowest quartile (Q1) to 59.17 years in the highest quartile (Q4). The proportion of men also increased, from 43.35% in Q1 to 51.83% in Q4. In terms of race/ethnicity, the proportion of Non-Hispanic White people was highest in Q4 (75.34%) compared to 56.18% in Q1. Educational attainment showed a slight decline with increasing ABSI, with the proportion of participants holding a college degree or higher falling from 34.75% in Q1 to 27.35% in Q4. Marital status also shifted, with the proportion of married individuals rising from 44.60% in Q1 to 58.09% in Q4. Regarding smoking and alcohol consumption, the proportion of former smokers increased significantly from 15.57% in Q1 to 34.54% in Q4, while the proportion of current drinkers remained relatively constant across the groups. In terms of physical activity, the proportion of inactive participants rose from 32.43% in Q1 to 56.73% in Q4, with a corresponding decrease in those engaging in vigorous exercise.

The prevalence of hypertension and diabetes also increased markedly with higher ABSI, reaching 47.89 and 19.15% in Q4, respectively, compared to 17.97 and 3.39% in Q1. These findings indicate significant differences in health status and lifestyle across ABSI quartiles, providing important context for subsequent analyses (see Table 1).

### 3.2 Association between a body shape index and depressive symptoms

This study examined the association between A Body Shape Index (ABSI) and depressive symptoms, as measured by PHQ-9 scores. Higher ABSI values were positively correlated with greater severity of depressive symptoms. In the multiple linear regression analysis, no significant differences were observed between groups in the unadjusted model. However, after stepwise adjustment for covariates—including age, sex, race, marital status, poverty income ratio, smoking status, alcohol consumption, physical activity, diabetes, and hypertension—higher ABSI was significantly associated with a greater risk of depressive symptoms. In the fully adjusted model (Adjusted II), participants in the third quartile (Q3) exhibited a significant increase in depressive symptoms compared with those in the first quartile (Q1) (*β* = 0.87, 95% CI = 0.68–1.06, *p* < 0.0001). This indicates that higher ABSI values were closely linked to greater depressive symptom severity. Similarly, the highest ABSI group (Q4) showed a significant association with depressive symptoms (*β* = 0.22, 95% CI = 0.02–0.41, *p* = 0.0323), as presented in Table 2. These findings underscore the increased risk of severe depressive symptoms in individuals with higher ABSI.

**Figure 2:**
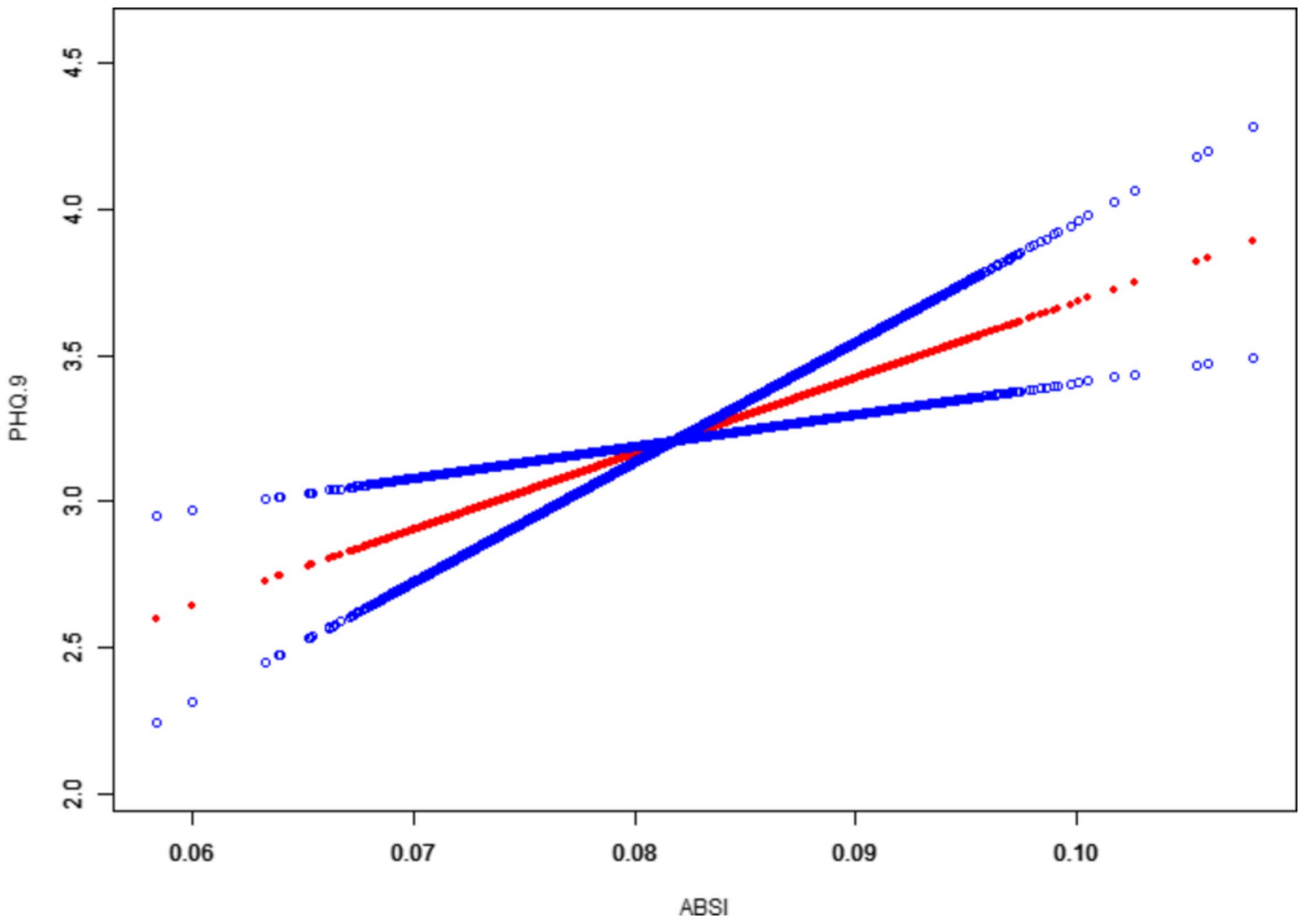
Association between ABSI and PHQ-9 scores with smoothed curve fitting.

Moreover, the smoothed curve fitting revealed a generally linear relationship between ABSI and PHQ-9 scores, with some non-linear tendencies observed at the extremes of ABSI values, as shown in Figure 2.

Depressive symptoms showed a steady increase with rising ABSI, particularly in the higher ABSI range. This suggests a strong association between an abnormally large waist circumference relative to height and BMI and the severity of depressive symptoms, especially in individuals with elevated ABSI. In conclusion, this study demonstrates that individuals with higher ABSI had significantly more severe depressive symptoms, even after adjusting for potential confounders such as sociodemographic and lifestyle factors. These results suggest that ABSI may serve as a useful indicator for assessing the risk of depressive symptoms and point to a complex interplay between body shape and mental health.

### 3.3 Subgroup analyses and interaction tests

Subgroup analyses were conducted to explore the variability of the association between ABSI and depressive symptoms across different populations, considering interactions with variables such as sex, race, age, educational level, poverty income ratio (PIR), smoking status, alcohol consumption, hypertension, diabetes, and marital status. After adjusting for all covariates, these analyses revealed patterns of association and potential interaction effects across subgroups (Table 3).

In the Crude model, no covariates were included, allowing for an unadjusted examination of the raw association between ABSI and depressive symptoms. In contrast, Model II adjusted for all covariates except for the subgroup variable of interest. For example, in the gender subgroup analysis, the model included covariates such as Age, Race, Education level, Marital status, High blood pressure, Diabetes, Physical activity, Smoking status, Alcohol status, and PIR, while excluding gender. This approach ensured that the observed associations reflected the independent effect of ABSI within each subgroup while accounting for potential confounding factors.

#### 3.3.1 Gender and ethnicity

In the gender subgroup analysis, both men (*β* = 29.24, 95% CI = 4.82–53.65, *p* = 0.0189) and women (*β* = 22.16, 95% CI = 3.03–41.30, *p* = 0.0232) showed significant positive associations between ABSI and depressive symptoms. However, the interaction effect of gender was not significant (*p* = 0.6509), indicating a minimal influence of gender on this association. Similarly, by race, higher ABSI was significantly associated with depressive symptoms in Non-Hispanic White people (*β* = 30.07, 95% CI = 6.18–53.96, *p* = 0.0136) and non-Hispanic Black (*β* = 31.14, 95% CI = 0.32–61.95, *p* = 0.0477) populations, with no significant interaction effect by race (*p* = 0.8767).

#### 3.3.2 Age and educational attainment

The association between ABSI and depressive symptoms was most pronounced among participants under 40 years of age (*β* = 27.07, 95% CI = 1.34–52.80, *p* = 0.0392), while no significant associations were observed in the 40–60 or 60+ age groups. Interaction analyses suggested a non-significant effect of age on the overall ABSI-depression association (*p* = 0.7608). For educational attainment, although the interaction effect was not significant (*p* = 0.4355), individuals with a college or associate’s degree demonstrated a stronger association (*β* = 46.01, 95% CI = 19.50–72.52, *p* = 0.0007), suggesting that higher educational levels may heighten sensitivity to the association between ABSI and depressive symptoms.

#### 3.3.3 Poverty income ratio, smoking, and drinking status

In the PIR subgroup, significant associations were found among individuals with PIR ≥1 and < 2 (*β* = 36.61, 95% CI = 7.72–65.50, *p* = 0.0130) and PIR ≥2 (*β* = 29.67, 95% CI = 8.24–51.11, *p* = 0.0067), while no associations were observed for PIR <1. Among current smokers (*β* = 53.40, 95% CI = 19.27–87.54, *p* = 0.0022) and current drinkers (*β* = 28.45, 95% CI = 11.48–45.42, *p* = 0.0010), ABSI was significantly associated with depressive symptoms, whereas weaker associations were observed among non-smokers and non-drinkers. Interaction analyses showed no significant interaction effects for smoking and drinking status (*p* = 0.1658 and *p* = 0.6898, respectively).

#### 3.3.4 Hypertension, diabetes, and marital status

For hypertension, the association between ABSI and depressive symptoms was significant in those without hypertension (*β* = 38.20, 95% CI = 18.94–57.45, *p* = 0.0001), but no significant association was observed in hypertensive individuals (*p* = 0.6379). Interaction analyses indicated a significant moderating effect of hypertension on this association (*p* = 0.0417), suggesting that hypertension may attenuate the relationship between ABSI and depressive symptoms. Diabetes status, however, did not show a significant interaction effect (*p* = 0.7883). Marital status analyses revealed significant associations among divorced (*β* = 79.80, 95% CI = 34.78–124.82, *p* = 0.0005), separated (*β* = 145.26, 95% CI = 67.77–222.74, *p* = 0.0002), and never married (*β* = 40.92, 95% CI = 6.78–75.07, *p* = 0.0188) individuals, while no significant differences were found for married or widowed participants. Interaction tests showed a significant moderating effect of marital status on the ABSI-depressive symptom relationship (*p* = 0.0004).

These subgroup analyses demonstrate that while gender, race, age, PIR, smoking/drinking status, hypertension, and marital status were evaluated for their potential moderating roles in the association between ABSI and depressive symptoms, only hypertension and marital status showed statistically significant interaction effects. This suggests that the relationship between ABSI and depressive symptoms may be more complex in these specific subgroups. These findings highlight the importance of a more nuanced understanding of ABSI’s effects on depressive symptoms across different populations, which could have implications for developing personalized interventions.

## 4 Discussion

### 4.1 Main findings

The aim of this study was to investigate the association between A Body Shape Index (ABSI) and depressive symptoms. The findings indicate that individuals with higher ABSI scores were more likely to exhibit more severe depressive symptoms. Higher ABSI was significantly associated with increased PHQ-9 depression scores after adjustment for covariates such as sociodemographic, lifestyle, and health factors, while the association was not significant in the unadjusted model. In the fully adjusted model (Model II), individuals in the Q3 and Q4 groups demonstrated a significantly higher risk of depressive symptoms, further validating the potential role of ABSI in assessing depressive symptoms. Subgroup analyses revealed variations in the association between ABSI and depressive symptoms across different populations. Distinct moderating effects were observed based on sex, race, age, income level, smoking and drinking status, marital status, and hypertension. Notably, hypertension and marital status showed particularly strong interactions with ABSI in relation to depressive symptoms, suggesting that complex mechanisms may underlie the relationship between physical form and mental health in specific groups. These findings, particularly in light of the growing focus on abdominal fat distribution and its influence on mental health, underscore the potential value of ABSI as a tool for assessing an individual’s risk of depression. They also provide a foundation for future studies aimed at further elucidating the relationship between body shape and mental health.

The results of this study align with previous research on the relationship between body mass index (BMI) and mental health (30, 31). Previous studies have demonstrated that waist circumference and abdominal obesity are strongly associated with negative mental health outcomes, including anxiety, depression, and heightened psychological stress (32, 33). ABSI, by better capturing the independent effects of waist circumference on health than traditional BMI, offers a novel perspective on the relationship between body shape and depressive symptoms. Unlike earlier studies that focused on BMI, this study extends the literature by analyzing the association between the emerging metric of ABSI and depressive symptoms.

Many previous studies have identified a positive relationship between higher BMI and depression (34, 35), but BMI is limited in its ability to distinguish between muscle and fat mass and fails to adequately reflect waist circumference, a key factor in health outcomes. In contrast, ABSI incorporates waist circumference, height, and BMI, offering a more nuanced measure of body shape and fat distribution. This study highlights the advantages of ABSI by demonstrating its stronger association with depressive symptoms compared to BMI, particularly in individuals with larger waist circumferences. Higher ABSI values may reveal hidden risks for depression, providing a more comprehensive assessment of mental health risks associated with abdominal obesity. Compared to waist circumference alone, ABSI adjusts for height and BMI, reducing potential confounding effects and offering a standardized representation of abdominal fat distribution. Unlike weight-adjusted waist indices, ABSI emphasizes the unique contribution of waist circumference to health outcomes while accounting for overall body size and proportions. This makes ABSI particularly valuable in identifying individuals at risk for depression, even when their BMI or raw waist circumference measurements fall within normal ranges. Consistent with prior research, this study also suggests that abdominal obesity may adversely affect mental health through mechanisms such as body dissatisfaction, social stigma, and endocrine dysfunction. The significant association between higher ABSI and depressive symptoms supports the hypothesis that an excessive increase in waist circumference, relative to height and BMI, may be a potential trigger for depressive symptoms.

Unlike studies that have primarily focused on overall adiposity (i.e., BMI) or isolated measures like waist circumference, this study underscores the unique role of ABSI in capturing the health impacts of abdominal fat distribution. These findings position ABSI as a valuable complementary indicator in mental health assessment, aligning with recent research trends that emphasize the importance of waist circumference and abdominal obesity in understanding mental health risks. Future research should explore the potential of ABSI in predicting other mental health outcomes, further elucidating the relationship between body shape and psychological well-being.

This study found a significant association between ABSI and depressive symptoms, with a notable increase in the severity of depressive symptoms, particularly in individuals with high ABSI. Several biological mechanisms and psychosocial factors may underlie this association, offering important avenues for further exploration.

First, abdominal fat and endocrine dysfunction likely play a crucial role. Visceral fat, in particular, is a key contributor to metabolic disorders such as insulin resistance and metabolic syndrome, as well as chronic inflammation (36). These conditions may affect the hypothalamic–pituitary–adrenal (HPA) axis, leading to increased secretion of stress hormones, such as cortisol (37). Chronic elevations in cortisol levels are strongly associated with depressive symptoms and are thought to contribute to the development of mood disorders, anxiety, and depression (38). Thus, the physiological basis for increased depressive symptoms may be linked to the association between increased waist circumference (high ABSI) and endocrine dysregulation. Second, chronic low-grade inflammation is another potential mechanism in the association between ABSI and depression. Visceral adipose tissue is a significant source of pro-inflammatory cytokines, including tumor necrosis factor-alpha (TNF-alpha) and interleukin-6 (IL-6) (39, 40). These inflammatory factors are not only linked to metabolic diseases but also to the onset and exacerbation of depressive symptoms. Chronic inflammation may impair mood regulation by altering neurotransmitter levels in the brain, a process that may be more pronounced in individuals with high ABSI.

Psychosocial factors also contribute to the relationship between ABSI and depressive symptoms. Individuals with larger waist circumferences often face greater social stigma and body size discrimination. Studies suggest that obese individuals may experience negative social evaluations, leading to body image dissatisfaction, lowered self-esteem, and social isolation—all of which are potential triggers for depression (41). Body image dissatisfaction is particularly influential in mental health outcomes, especially among women and adolescents (42). Larger waist circumferences may have a greater impact on emotional well-being in these populations. Additionally, physical discomfort and reduced mobility associated with a higher ABSI may contribute to depressive symptoms. A larger waist circumference can limit physical activity and reduce quality of life (43). Physical inactivity is not only associated with poor physical health but can also affect mental health by reducing the release of endorphins, thereby increasing the risk of depressive symptoms (44). Finally, a negative feedback loop may exist between ABSI and depressive symptoms, exacerbated by psycho-physical interactions. Depressive symptoms can lead to unhealthy eating habits and decreased physical activity, contributing to the accumulation of abdominal fat and further increasing ABSI. This vicious cycle of worsening depressive symptoms and deteriorating body shape may intensify mental health challenges.

Notably, hypertension showed a particularly strong interaction with ABSI in relation to depressive symptoms, suggesting that this condition may amplify the relationship between physical form and mental health through complex mechanisms. Chronic hypertension is associated with systemic inflammation and dysregulation of the hypothalamic–pituitary–adrenal (HPA) axis, both of which have been implicated in the pathogenesis of depression (45). Elevated levels of pro-inflammatory cytokines, such as interleukin-6 (IL-6) and tumor necrosis factor-alpha (TNF-*α*), may exacerbate the adverse psychological effects of body composition (46). Furthermore, hypertension imposes a psychological burden, including heightened stress and anxiety, which could further contribute to depressive symptoms (47). Behaviorally, individuals with hypertension often experience reduced physical activity levels, diminishing a key protective factor against depression and potentially compounding the effects of ABSI.

In conclusion, multiple biological mechanisms and psychosocial factors likely interact to form the association between ABSI and depressive symptoms. Future studies should further investigate the specific pathways underlying these mechanisms and consider the bidirectional effects of body shape and mental health in developing interventions aimed at preventing and managing depressive symptoms more effectively.

### 4.2 Strengths and limitations

This study has several strengths. First, the use of ABSI as an indicator of body shape offers a novel approach, distinct from the traditional reliance on BMI. ABSI more accurately reflects the unique impact of waist circumference relative to height and BMI, effectively capturing health risks associated with visceral fat distribution. This makes it particularly valuable for assessing the relationship between body shape and mental health, enabling a more targeted analysis. Second, the study draws on data from the National Health and Nutrition Examination Survey (NHANES), a nationally representative survey with a large and diverse sample that encompasses a wide range of sociodemographic characteristics. This ensures that the findings are generalizable to the broader U.S. population. Moreover, the study employed a multivariate adjustment model to control for potential confounders, including sociodemographic factors, lifestyle, and health status, enhancing the robustness of the results.

However, there are limitations to this study. First, the cross-sectional design precludes the establishment of a causal relationship between ABSI and depressive symptoms. While we identified a significant association, we cannot infer whether elevated ABSI directly causes depressive symptoms. Future longitudinal studies or randomized controlled trials are needed to validate the causality of this relationship. Second, depressive symptoms were assessed using the self-reported PHQ-9 scale. Although widely used, self-report measures can introduce bias and may underestimate the severity of depression. Similarly, the calculation of ABSI relies on accurate measurements of waist circumference, height, and BMI. Although NHANES provides high-quality anthropometric data, potential measurement errors in other datasets or self-reported data could affect the accuracy and consistency of ABSI assessments. Another limitation is the study’s focus on a single body shape indicator—abdominal fat. While ABSI provides a more nuanced assessment of waist circumference, it does not account for other body fat distributions (e.g., hip circumference, waist-to-hip ratio), which may also influence mental health. Future research should incorporate multiple body shape indicators to offer a more comprehensive evaluation of their potential roles in depressive symptoms. Lastly, despite adjusting for several covariates, there may still be unmeasured confounders, such as dietary habits, sleep quality, and social support, that could influence the association between ABSI and depressive symptoms. Future studies could further refine the analysis by accounting for these factors.

In conclusion, this study provides new evidence for the association between ABSI and depressive symptoms. However, the aforementioned limitations should be considered when interpreting the results. Future research should incorporate a broader range of body image indicators and longitudinal designs to further explore the complex relationship between body shape and mental health.

## 5 Conclusion

This study systematically examined the association between A Body Shape Index (ABSI) and depressive symptoms, revealing that higher ABSI scores were significantly associated with more severe depressive symptoms after adjusting for a range of sociodemographic, lifestyle, and health-related covariates. These findings suggest that the independent contribution of waist circumference relative to height and BMI may be an important predictor of depressive symptoms. By employing ABSI as a tool for body shape assessment, we were able to gain deeper insights into the potential impact of abdominal fat distribution on mental health.

## Data Availability

The original contributions presented in the study are included in the article/supplementary material, further inquiries can be directed to the corresponding author.
